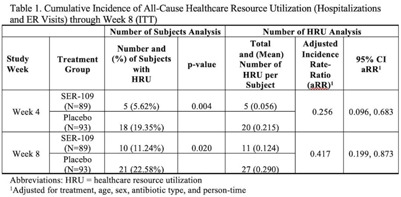# Healthcare resource utilization in a phase 3 trial of SER-109 in patients with recurrent Clostridioides difficile infection

**DOI:** 10.1017/ash.2022.196

**Published:** 2022-05-16

**Authors:** Stuart Cohen, Thomas Louie, Charles Berenson, Alpesh Amin, David Lombardi, Sissi Pham, Shirley Huang, Elaine Wang, Brooke Hasson, Barbara McGovern, Lisa Von Moltke

## Abstract

**Background:** The estimated economic cost of *Clostridioides difficile* infection (CDI) is $5.4 billion annually, primarily attributed to acute-care costs. We previously reported data from ECOSPOR III that SER-109, an investigational oral microbiome therapeutic, was superior to placebo in reducing recurrent CDI (rCDI) in adults at 8 weeks after treatment, with a 68% relative risk reduction. Adults with rCDI have more hospitalizations and emergency room (ER) visits (defined herein as healthcare resource utilization, HRU) compared to those without recurrence. Thus, we evaluated incidence of HRU. **Methods:** Adults with rCDI (≥3 episodes in 12 months) were screened at 56 US and Canadian sites and were randomized 1:1 to SER-109 (4 capsules × 3 days) or placebo following resolution of CDI with standard-of-care CDI antibiotics. The primary end point was rCDI at 8 weeks. Exploratory end points included cumulative incidence of hospitalizations through 24 weeks after treatment. Here, we report cumulative incidence of all-cause HRU through 8 weeks after treatment. **Results:** In total, 281 patients were screened and 182 were randomized (59.9% female; mean age 65.5 years; 98.9% outpatient). Overall, 31 patients (17%) had 38 hospitalizations or ER visits through week 8 (11 events in 10 SER-109 patients and 27 events in 21 placebo patients) (Table 1). The cumulative incidence of HRU was lower in SER-109–treated patients compared to placebo at both weeks 4 and 8 with most events (65.8%) recorded within 4 weeks after treatment. The adjusted HRU incidence rate (by person time, age, sex, and antibiotic use) was also lower in SER-109–treated patients compared to placebo at weeks 4 and 8 (0.256 [95% CI, 0.096–0.683] versus 0.417 [95% CI, 0.199–0.873], respectively). **Conclusions:** SER-109–treated patients had less HRU compared to placebo patients through 8 weeks after treatment in this mostly outpatient population. These data suggest a potential benefit of SER-109 in reducing HRU, thus lowering the healthcare burden of rCDI.

**Funding:** Seres Therapeutics

**Disclosures:** None

**Table:**